# Rapid Extraction and Identification of Maitotoxin and Ciguatoxin-Like Toxins from Caribbean and Pacific *Gambierdiscus* Using a New Functional Bioassay

**DOI:** 10.1371/journal.pone.0160006

**Published:** 2016-07-28

**Authors:** Richard J. Lewis, Marco Inserra, Irina Vetter, William C. Holland, D. Ransom Hardison, Patricia A. Tester, R. Wayne Litaker

**Affiliations:** 1 Institute for Molecular Bioscience, The University of Queensland, Brisbane, 4072, Australia; 2 National Oceanic and Atmospheric Administration, National Ocean Service, Center for Coastal Fisheries & Habitat Research, 101 Pivers Island Road, Beaufort, NC, 28516, United States of America; University of Wisconsin Milwaukee, UNITED STATES

## Abstract

**Background:**

Ciguatera is a circumtropical disease produced by polyether sodium channel toxins (ciguatoxins) that enter the marine food chain and accumulate in otherwise edible fish. Ciguatoxins, as well as potent water-soluble polyethers known as maitotoxins, are produced by certain dinoflagellate species in the genus *Gambierdiscus* and *Fukuyoa* spp. in the Pacific but little is known of the potential of related Caribbean species to produce these toxins.

**Methods:**

We established a simplified procedure for extracting polyether toxins from *Gambierdiscus* and *Fukuyoa* spp. based on the ciguatoxin rapid extraction method (CREM). Fractionated extracts from identified Pacific and Caribbean isolates were analysed using a functional bioassay that recorded intracellular calcium changes (Ca^2+^) in response to sample addition in SH-SY5Y cells. Maitotoxin directly elevated Ca^2+^_i_, while low levels of ciguatoxin-like toxins were detected using veratridine to enhance responses.

**Results:**

We identified significant maitotoxin production in 11 of 12 isolates analysed, with 6 of 12 producing at least two forms of maitotoxin. In contrast, only 2 Caribbean isolates produced detectable levels of ciguatoxin-like activity despite a detection limit of >30 pM. Significant strain-dependent differences in the levels and types of ciguatoxins and maitotoxins produced by the same *Gambierdiscus* spp. were also identified.

**Conclusions:**

The ability to rapidly identify polyether toxins produced by *Gambierdiscus* spp. in culture has the potential to distinguish ciguatoxin-producing species prior to large-scale culture and in naturally occurring blooms of *Gambierdiscus* and *Fukuyoa* spp. Our results have implications for the evaluation of ciguatera risk associated with *Gambierdiscus* and related species.

## Introduction

Ciguatera is a common marine poisoning caused by the consumption of tropical and sub-tropical fishes contaminated with potent polyether channel toxins known as ciguatoxins [1]. Ciguatoxins activate voltage sensitive sodium channels (VSSC) and certain potassium channels to produce a range of long-lasting gastrointestinal and neurological symptoms, including the pathgnomonic symptom of reversal of temperature perception or cold allodynia [2]. Ciguatoxins are produced by *Gambierdiscus* and *Fukuyoa* spp. (unpublished data), a group of benthic dinoflagellates grazed on by herbivorous fishes and invertebrates. Following blooms, the less oxidized ciguatoxins are biotransformed and accumulated as they transfer through marine food chains to carnivorous fishes [3]. Presently, apart from not eating risk species, there is no simple way to avoid consuming ciguateric fish.

A Japan-French expedition to the Gambier Islands first identified a benthic dinoflagellate bloom that produced ciguatoxin-like toxins and was the likely origin of ciguatera. The anterior-posteriorly compressed (discoid shaped) microalga dominating this bloom was later named *Gambierdiscus toxicus* [4, 5]. However, detailed genetic and morphological comparisons now suggest the bloom was comprised of a mix of morphologically similar species [6]. To date, eleven anterior-posteriorly compressed species have been described (*Gambierdiscus australes* M Chinian & MA Faust, *G*. *belizeanus* MA Faust, *G*. *caribaeus* Litaker, Faust, Kibler, Holland & Tester, *G*. *carolinianus* Litaker, Vandersea, Faust, Kibler, Holland & Tester, *G*. *carpenteri* Kibler, Litaker, Faust, Holland, Vandersea & Tester, *G*. *excentricus* S Fraga, G. silvae S Frag, F Rodríguez, *G*. *pacificus* M Chinain, M Faust, *G*. *polynesiensis* M Chinain, M Faust, *G*. *scabrosus* T Nishimura, S. Sato, M Adachi, *G*. *toxicus* R Adachi, Y Fukuyo) [6–11]. The two described globular *Gambierdiscus* species, *G*. *yasumotoi* (MJ Holmes) [12] and *G*. *ruetzleri* (Faust, Litaker, Vandersea, Kibler, Holland & Tester) [6], were recently transferred to the newly described genus *Fukuyoa* (*F*. *ruetzleri* (F Gómez, D Qiu, RM Lopes, S Lin); *F*. *yasumotoi* (F.Gómez, D Qiu, RM Lopes & S Lin), based on cell morphology and molecular phylogenetic evidence [13]). At the same time, a new type species for the genus, *F*. *paulensis* (F Gómez, D Qiu, RM Lopes, S Lin) was described, with the species present varying depending on location [14]. This diversity, together with strain-dependent variations in toxin production, likely explain the variable occurrence of distinct classes of ciguatoxins (CTX) found in fishes in the Pacific Ocean (P-CTX), the Indian Ocean (I-CTX) and the Caribbean Sea (C-CTX) [15]. At least three forms of MTX are also produced by *Gambierdiscus* and related species but these have not been shown to accumulate to significant levels in the flesh of fish [3].

To better understand the levels and types of polyether toxins produced by different *Gambierdiscus* spp., we developed a simplified extraction procedure to isolate toxins present in *Gambierdiscus* samples. The bioactivity of these samples was assessed using a SH-SY5Y cell-based FLIPR^®^ assay (Molecular Devices, Sunnyvale, CA) that measured toxin-induced calcium influx. SH-SY5Y cells are human neuroblastoma cells that endogenously express tetrodotoxin-sensitive voltage-gated sodium channel (Na_V_) isoforms as well as a range of Ca^2+^ channels [16, 17]. While we have previously described that purified ciguatoxins induce Ca^2+^ responses in these cells through activity at endogenously expressed Na_V_ channels [18], optimization of assay conditions for detection of both purified ciguatoxins and ciguatoxin-containing extracts has not been reported. In addition, a direct comparison to commonly used cytotoxicity assays using the murine neuroblastoma cell line Neuro2a is lacking. We thus established concentration-response curves of purified P-CTX-1, P-CTX-2 and P-CTX-3 to determine potentiation of veratridine-induced responses. Our SH-SY5Y Ca^2+^ assay performed comparable to a Neuro2a-based cytotoxicity assay and was used to analyze 12 isolates of *Gambierdiscus* spp. obtained from the Pacific Ocean and Caribbean Sea. Our approach revealed significant strain and species differences in the levels and types of ciguatoxins and maitotoxins produced by *Gambierdiscus* spp.

## Methods

### Reagents

All reagents used in this study were ACS grade or higher. Solvents were HPLC grade or higher purity. All water used was Milli-Q Ultra-pure grade with 18.2 MΩ resistivity. All reagents used in growing cells and for extracting CTX and MTX were purchased from Sigma Aldrich, St. Louis, Missouri. Reagents and solvents used for fractionation of *Gambierdiscus* extracts and measurement of CTX- and MTX-like activity using FLIPR assay were purchased from Sigma Aldrich, Castle Hill, NSW, Australia unless otherwise stated. P-CTX-1, P-CTX-2 and P-CTX-3 standards were isolated and purified from *Gymnothorax javanicus* liver, as previously described [19].

### *Gambierdiscus* Collection, Identification and Culture

The *Gambierdiscus* isolates screened in this study were obtained either from the National Centre for Marine Algae and Microbiota (East Boothbay, Maine, USA; formally CCMP) or from single cell isolates collected at locations throughout the Caribbean, Gulf of Mexico or tropical Pacific (Table 1). Single cell isolates were obtained as follows. Using either a high power stereoscope or inverted microscope, a series of *Gambierdiscu*s cells were drawn into a Pasteur pipette and transferred to a 8 well Petri dish containing one ml of filter-sterilized sea water (0.2 μm Meissner Filtration Products, Inc. STyLux, Bodenheim Germany, cat. No. cssm 0.2.-222). A total of between 10 and 20 cells were added to each well. This pre-isolation step helped dilute any contaminating cells. Next, individual cells were sequentially transferred through three to four drops of filter-sterilized seawater to ensure a single cell was present. Each cell was then added to a separate well in a 24 well plate that was approximately half-filled with filter-sterilized medium. A few drops of Modified K medium (without ammonium, copper or TRIS; [20]) was then added to each of the wells. The culture plate was sealed with paraffin film (or placed in a Ziploc^®^ bag (S. C. Johnson & Son, Racine, Wisconsin) to limit evaporation and incubated at low light (30–50) μmol photons m^-2^ s^-1^ at 27°C, a temperature similar to that prevailing at the sample collection sites. Cell growth was monitored every 2–3 days using an inverted microscope. Once the isolated cells had completed several division cycles, the entire volume of each well was transferred to a larger vessel containing dilute growth medium (~25% full strength growth medium). As the cultures were sequentially transferred, the proportion of medium was increased until it reached 100%, allowing cells to gradually acclimate to the artificial medium.

**Table 1:** Isolates of *Fukuyoa* and *Gambierdiscus* spp. from the Pacific and Caribbean and number of CTX- and MTX-like congeners detected using the cell-based assay. The + indicates broad peak(s) likely contain more than a single MTX congener.

Species	Strain	Cells fractionated	Location isolated	Basin	CTXs	MTXs
*F*. *ruetzleri*	Gam1	1.7 x 10^6^	Southwater Caye, Belize	Caribbean	3 (3)^a^	1+
*G*. *australes*	W B Gam 3	1.7 x 10^6^	Waikiki Beach, Hawaii, USA	Pacific	0	2+ (1)^a^
*G*. *belizeanus*	CCMP 399	0.9 x 10^7^	St. Barthelemy, Collectivity of France	Caribbean	(4)^a^	4+
*G*. *caribaeus*	Gam 19	1.3 x 10^6^	Carrie Bowe Caye Belize	Caribbean	0	(3)^a^
*G*. *caribaeus*	Pat HI Jar 2 Gam 2	1.1 x 10^6^	Big Island, Hawaii, USA	Pacific	0	3+
*G*. *carpenteri*	GT4	1.4 x 10^6^	Carrie Bowe Cay, Belize, USA	Caribbean	0	3+
*G*. *carpenteri*	Pat HI Jar 7 Gam 11	1.1 x 10^6^	Hawaii, USA	Pacific	0	3+
*G*. *carolinianus*	Kenny 6	1.1 x 10^6^	Offshore North Carolina, USA	Atlantic	0	3
*G*. *carolinianus*	Dive 1 Gam 1	1.2 x 10^6^	Carrie Bowe Caye, Belize	Caribbean	2	2
*G*. *carolinianus*	Pat HI Jar 3 Gam 9	1.2 x 10^6^	Big Island, Hawaii, USA	Pacific	0	3+
*Gambierdiscus* ribotype 2	St. Maartens Gam 6	2.6 x 10^6^	St. Maartens, Collectivity of France	Caribbean	2 (1)^a^	2+
*Gambierdiscus* ribotype 2	Mixed PR Gam 3	1.4 x 10^6^	Puerto Rico, USA	Caribbean	1 (2)^a^	2+

() ^a^ Number of potential congeners present at low levels are shown in brackets

Once adapted to full strength Modified K medium, cells were acclimated to grow at 27°C, 100 μmol photons m^-2^ s^-1^, 14:10 light:dark cycle, which has been shown previously to provide near optimal growth in all the species tested [21]. Changes in cell density were monitored every third day using a Coulter Counter Multisizer 3 particle counter with a 280 μm aperture and 1 ml sample volume (Beckman Coulter, Brea California, USA). When the cells reached early stationary phase, they were counted a final time using the Coulter Counter and concentrated onto a 20 μm sieve. Cells were then washed with seawater into a 50 ml glass centrifuge tube. The cells were pelleted at 3200 x g for 10 min and the supernatant was carefully removed. The final pellets were either processed immediately or stored at –80°C prior to extraction.

### Extraction of *Gambierdiscus* Cells

Cell pellets containing 1–3 million cells were sonicated twice for 1 min in methanol:water:hexane (2:1:1) at 10 ml per 1 million cells using a Qsonica, Q700 unit (Thermo Fisher Scientific Inc., Waltham, Massachusetts) with the tip setting set at amplitude 50. Once the cells were disrupted, the sample was centrifuged at 3200 x g for 10 min and the supernatant transferred to a clean separatory funnel. The remaining pellet was resuspended in 10 ml of methanol:water:hexane (2:1:1), sonicated 2 X 1 min, centrifuged at 3200 x g for 10 min, and the supernatant added to the same separatory funnel. The hexane layer was discarded and the remaining methanol:water solution dried under N_2_ gas. The dried residue was then redissolved in 10 ml of dichloromethane (DCM), placed into a clean separatory funnel, and extracted twice with 5 ml of 60% aqueous methanol. The methanol:water fraction containing MTX and the DCM fraction containing CTX were collected in separate 20 ml scintillation vials and dried under ultra-high purity nitrogen.

### Fractionation of *Gambierdiscus* Extracts

The dried MTX and CTX extracts containing the equivalent of 1.1–9 x 10^6^ cells were resuspended in 0.5 ml of 30% acetonitrile (ACN) and fractionated on a Vydac 218TP C18 column (250 x 4.6 mm, 5 μm; Phenomenex (Torrence, California, USA)) eluted at 0.7 mL min^-1^ initially for 5 min at 5% acetonitrile (ACN)/0.1% formic acid and then with a linear gradient from 5–90% ACN/0.1% formic acid over 60 min. 70 x 1-min fractions were collected and 25% (v/v) of these fractions were freeze dried and resuspended in 15 μl physiological salt solution (PSS; composition NaCl 140 mM, glucose 11.5 mM, KCl 5.9 mM, MgCl_2_ 1.4 mM, NaH_2_PO_4_ 1.2 mM, NaHCO_3_ 5 mM, CaCl_2_ 1.8 mM, HEPES 10 mM) containing 0.1% bovine serum albumin (BSA) just prior to FLIPR assay analysis.

### Measurement of CTX- and MTX-like Activity Using Intracellular Ca^2+^ Responses

SH-SY5Y are human-derived neuroblastoma cells that express a range of voltage-gated sodium channel subtypes [17]. Use of these cells allows sensitive direct detection of MTX-induced calcium influx and indirect detection of CTX activity by measuring enhancement of veratridine-induced calcium influx [17]. Briefly, SH-SY5Y cells (ECACC, Salisbury, Wiltshire, UK) were maintained at 37°C/5% CO_2_ in RPMI media containing 15% fetal bovine serum (FBS) and 2 mM L-glutamine [17]. Cells were routinely passaged at a 1:5 dilution every 3–5 days using 0.25% trypsin/ethylenediaminetetraacetic acid (EDTA) (Gibco). SH-SY5Y cells were seeded at 120,000 cells/well in 40 μL of culture medium on black-walled 384-well imaging plates (Corning, Australia, #CLS3712) and cultured until 90–95% confluent monolayers were obtained (~48 h). Cells were then loaded for 30 min at 37°C with 20 μL of Calcium-4 No-Wash dye (#R8141, Molecular Devices, Sunnyvale, CA) in PSS containing 0.1% BSA. During this 30 min incubation period the dye is absorbed into the cells’ cytoplasm. Plates containing the loaded cells were then transferred to the FLIPR^TETRA^ (Molecular Devices, Sunnyvale, CA) fluorescent plate reader with a 470–495 nM excitation filter and 515–575 nM emission filter. Camera gain and intensity were adjusted so that each plate containing loaded cells yielded a minimum baseline fluorescence of 1000 arbitrary fluorescence units (AFU).

A two-addition protocol was used to determine activity of purified P-CTX-1, P-CTX-2 and P-CTX-3 (30 fM– 100 nM) as well as fractionated *Gambierdiscus*/*Fukuyoa* extracts. After a 10 s baseline measurement was recorded, 10 μl of sample was added to each well containing the loaded cells using a FLIPR^TETRA^ injection manifold. Fluorescence was recorded every s for 300 s thereafter. A second addition (10 μl) of buffer (PSS) or varying concentrations of veratridine (1, 3 and 5 μM) followed by a further 300 reads (1 read/s) was used to determine synergistic effects on Na_V_ activity.

To compute responses induced by MTX-like activity, responses were normalized to baseline (read 1–10) and the maximum increase in fluorescence for reads 10–300 determined using ScreenWorks 3.2.0.14 (Molecular Devices). For P-CTX and CTX-like activity, the maximum increase in AFU over baseline (reads 300–310) was determined for reads 310–600. Results were normalized by determining the peak AFU for each CTX fraction and normalizing it to the maximal AFU (100% activity) across all of the CTX fractions tested. To establish the relative levels of MTX in the absence of a suitable standard, results were normalized to the *Gambierdiscus/Fukuyoa* extract that produced the largest increase in AFU (100% activity) of the samples tested. Each fraction from both MTX and CTX was measured in triplicate.

### Cytotoxicity Assay

To compare the sensitivity of the calcium-based assay for CTX with the cytotoxicity assay developed by [18], the Neuro2a neuroblastoma (N2a) assay was run using the same 30 fM–100 nM P-CTX-1,2 and 3 standards used to establish the sensitivity of the FLIPR assay. Briefly, mouse Neuro2a neuroblastoma cells (American Type Culture Collection, Manassas, VA, USA) were maintained at 37°C/5% CO_2_ in Dulbecco’s Modified Eagle’s Medium (DMEM, SIGMA D0572) containing 10% heat-inactivated FBS, 2 mM L-glutamine, pyridoxine and 110 mg/ml sodium pyruvate. Cells were split every 4–6 days or when ~90% confluent at a 1:8 dilution using 0.25% trypsin/EDTA. For each assay, cells were seeded at 10,000 cells in 100 μl of culture medium for each well in a 96-well tissue culture plate (Corning, CLS3596). The plate was then incubated for ~24 h at 37°C/5% CO_2_ until wells were >90% confluent before 10 μl of P-CTX-1 (30 fM–100 nM) and 10 μl of medium containing 10 μM veratridine and 100 μM of ouabain were added and cells incubated for 24 h. Cell medium was then removed by pipette and replaced with DMEM solution containing thiazole blue tetrazolium bromide (Sigma-Aldrich, Castle Hill, Australia). Cells were incubated a further 45 min before dye removal. DMSO was then added to dried wells to resuspend the internalised dye, which was measured using a plate reader at 570 nm, with background at 630 nm subtracted.

## Results

### SH-SY5Y Cell Assay for CTX and MTX

**Fig 1:**
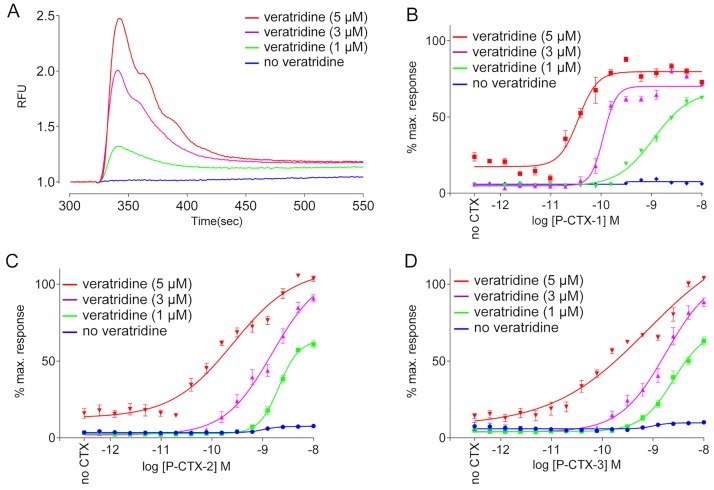
Synergistic effects of P-CTX-1, 2, 3 on veratridine responses in SH-SY5Y cells. (A) Representative veratridine-induced responses in SH-SY5Y cells pre-incubated for 300 s with 0.5 nM P-CTX-1. Blue trace, response in the absence of veratridine; green trace, response elicited by 1 μM veratridine; purple trace, response elicited by 3 μM veratridine; red trace, response elicited by 5 μM veratridine. (B-D) P-CTX-1, P-CTX-2 and P-CTX-3 (30 fM–100 nM) were added 300 s prior to addition of 1 μM (green), 3 μM (purple) or 5 μM (red) veratridine. The blue data in each panel show the response to P-CTX-1, 2 and 3 in the absence of veratridine. (B) Concentration-dependent potentiation of veratridine responses by P-CTX-1 (30 fM–100 nM). (C) Concentration-dependent potentiation of veratridine responses by P-CTX-2 (30 fM–100 nM). (D) Concentration-dependent potentiation of veratridine responses by P-CTX-3 (30 fM–100 nM). Data are presented as mean ± SEM from at least n = 3 wells and are representative of three independent experiments.

**Fig 2:**
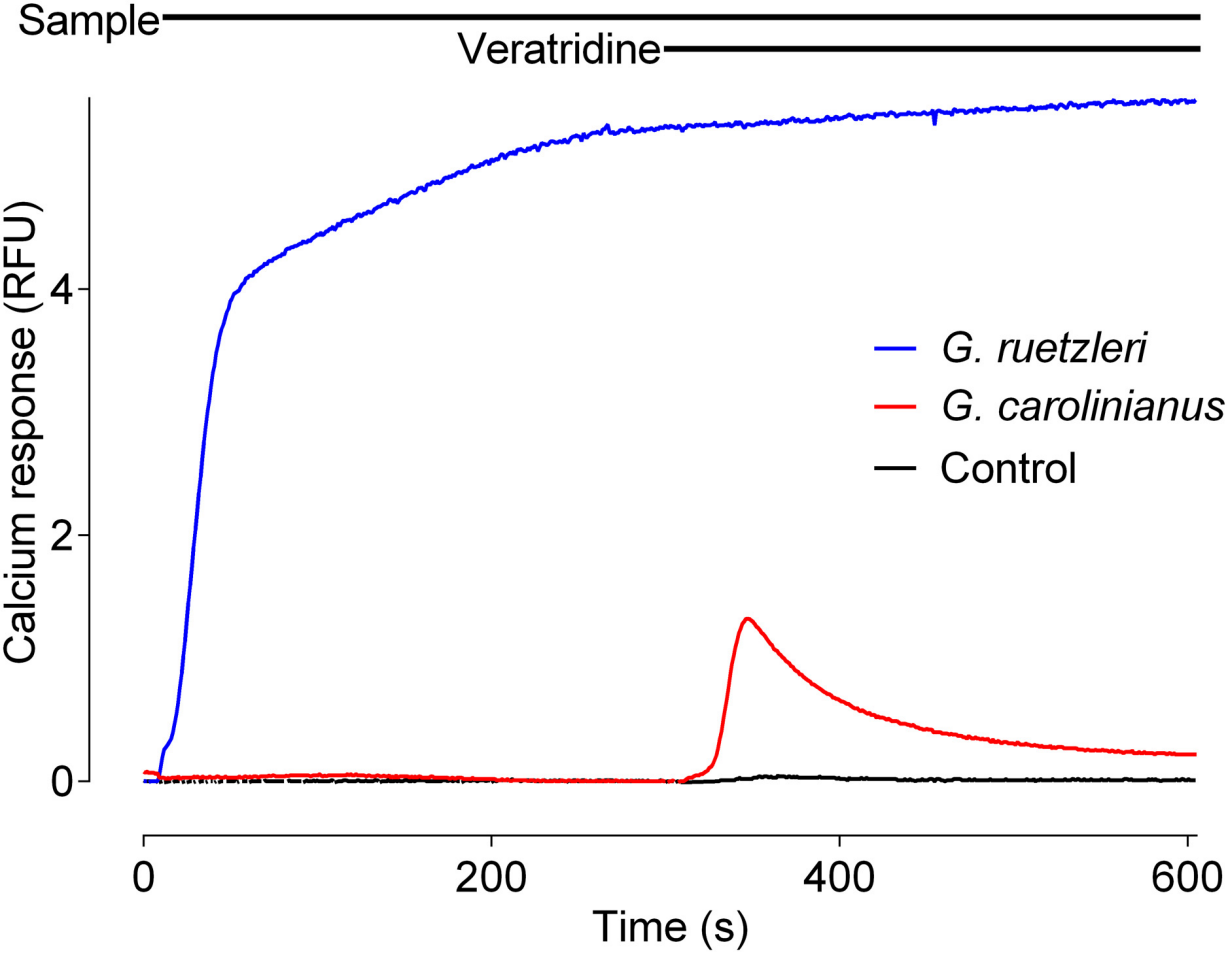
Fluorescence response of SH-SY5Y cells to MTX-like or CTX-like activity. Fluorescent responses to addition of HPLC fractionated samples was measured in SH-SY5Y cells loaded with Calcium-4-NW dye using a two-addition protocol. The presence of MTX-like activity (blue trace, *Fukuyoa ruetzleri* methanol fraction) caused a rapid increase in fluorescence that was maintained for the 600 s time course. In contrast, addition of samples containing CTX-like activity (red trace, *Gambierdiscus carolinianus* DCM fraction) caused no measurable increase in fluorescence until addition of veratridine (5 μM) after 300 s due to potentiation of veratridine-induced effects on endogenous Na_V_ channels by CTX-like activity. The resultant Ca_2+_ transient is proportional to the toxicity of CTX-like compounds present. The black trace indicates the buffer control response on the first addition as well as the veratridine response (5 μM) on second addition in the absence of pre-treatment with CTX.

To determine the optimal concentration of veratridine for the assay, cells loaded with Calcium-4 No-Wash dye were incubated for 300 s with 0.5 nM P-CTX-1 prior to adding different concentrations of veratridine. By itself, 1–5 μM veratridine had little effect on Ca^2+^_i_, however, pre-exposure to CTXs for 300 s significantly enhanced Ca^2+^_i_ in response (Fig 1A–1D). Maximum enhancement of the sensitivity of the assay for CTX was observed at 5 μM veratridine and this concentration was used to assess CTX-like activity in *Gambierdiscus*/*Fukuyoa* extracts. The MTX assay procedure was therefore extended to include a measure of CTX-like activity using veratridine addition 300 s after fraction addition (Fig 2). Samples containing measurable CTX fluorescence produced veratridine responses that peaked ~20 s after veratridine addition before slowly declining over ~100 s.

Although CTX also directly increases Ca^2+^_i_ at low nM concentrations [18], the level of sensitivity was insufficient to directly detect the low levels of CTX-like toxins typically present in the fractionated *Gambierdiscus*/*Fukuyoa* extracts. Since veratridine can enhance SH-SY5Y cell FLIPR responses to other sodium channel activator toxins [17], we investigated its potential to sensitize FLIPR responses. As previously described [17], veratridine concentrations of 5 μM produce a small but significant calcium response alone (“no CTX”, Fig 1B–1D). In contrast, between 1 and 5 μM veratridine, there was dose-dependently enhanced fluorescence as describe previously [17] (Fig 1A). The same enhancement of fluorescence was observed when cells were incubated with 30 fM to 100 nM of P-CTX-1, -2 and -3 for 300 seconds prior to addition 1, 3 or 5 μM veratridine (Fig 1B–1D). P-CTX-1 was the most potent isoform tested irrespective of the veratridine concentration. In the presence of 5 μM veratridine, the P-CTX-1 pEC_50_ was 10.04 ± 0.4, whereas P-CTX-2 and 3 had pEC_50_ values of 9.67 ± 0.06 and 9.35 ± 0.15, respectively (Table 1). Lower concentrations of veratridine produced less ciguatoxin potentiation for P-CTX-1, -2 and -3 (Fig 1A–1C; Table 2). Thus, given the strong potentiation of responses by P-CTX-1, P-CTX-2 and P-CTX-3 (Fig 1B, 1C and 1D), 5 μM veratridine was used to identify CTX-like activity in HPLC-fractionated extracts of *Gambierdiscus* spp. (Fig 2). In this format, the limit of detection was estimated to be >1 pg P-CTX-1 equivalents per well (40 μl). It should also be noted that in this format, MTX carry over into a given CTX fraction could interfere with the corresponding veratridine enhanced CTX measurement.

**Table 2:** Potency (pEC_50_ ± SEM) of P-CTX-1, -2 and -3 to potentiate veratridine responses in SH-SY5Y cell FLIPR assay (n = 3).

Ciguatoxin	Veratridine (μM)			
	0	1	3	5
P-CTX-1	inactive	8.95 ± 0.04	9.91 ± 0.06	10.04 ± 0.04
P-CTX-2	inactive	8.72 ± 0.1	8.91 ± 0.12	9.67 ± 0.06
P-CTX-3	inactive	8.74 ± 0.07	8.91 ± 0.08	9.35 ± 0.15

### Neuro2a assay for CTX

**Fig 3:**
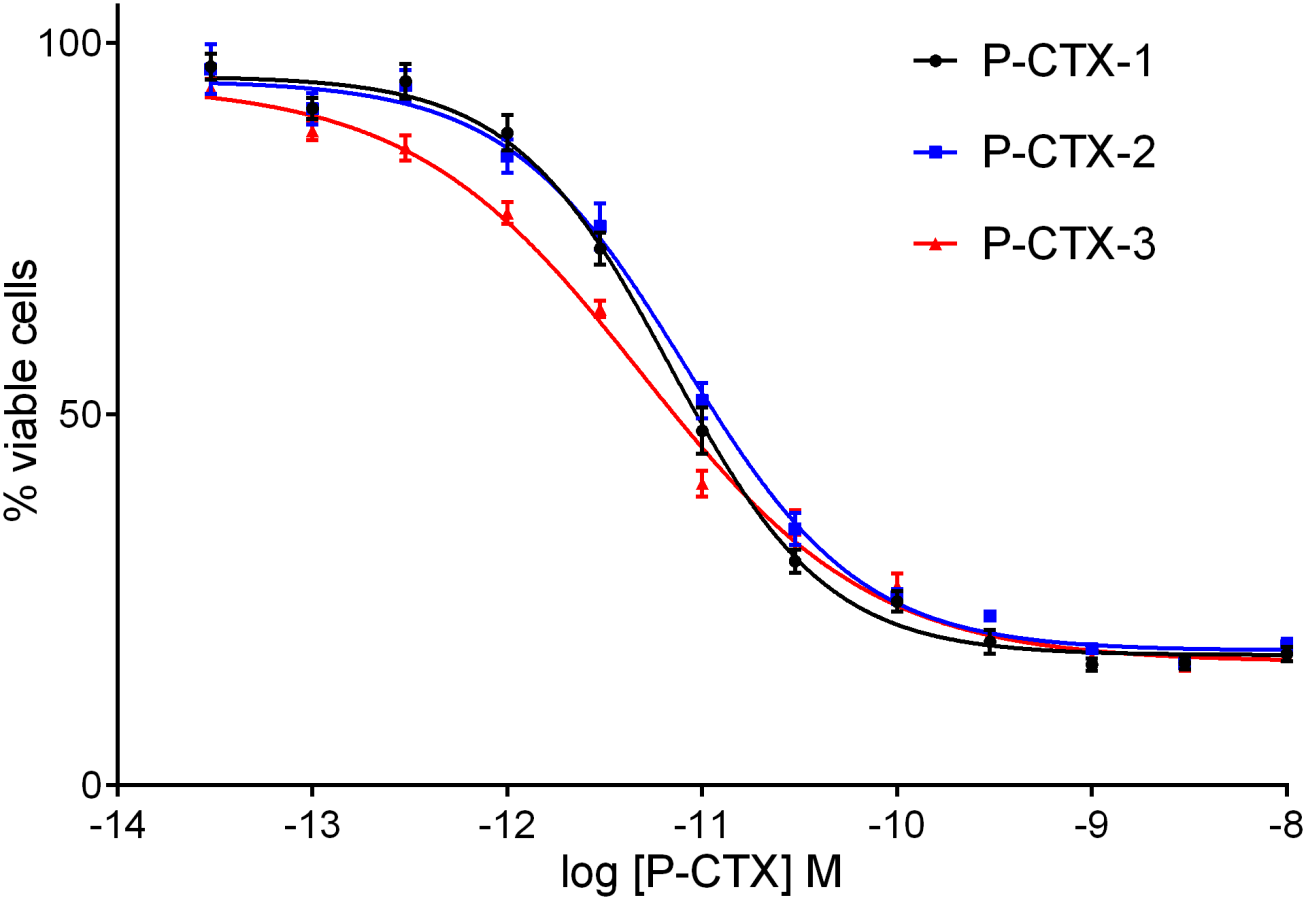
Cell viability assay. Cell death measured in Neuro2a cells treated with veratridine (10 μM), ouabain (100 μM) and varying concentrations of Pacific ciguatoxins. The pEC_50_ values of P-CTX-1, -2 and -3 were 11.17 ± 0.03, 11.09 ± 0.04 and 11.31 ± 0.04, respectively. Data are presented as value ± standard error (n = 3).

Neuro2a assays were performed to determine the relative sensitivity of the FLIPR assay versus the established Neuro2a neuroblastoma MTT cytotoxicity assay [22, 23]. The pEC_50_ values of P-CTX-1, -2 and -3 were 11.17 ± 0.03, 11.09 ± 0.04 and 11.31 ± 0.04, respectively. The limit of sensitivity was ~1 pg P-CTX-1 equivalents per well, similar to that exhibited by the FLIPR assay (Figs 1 and 3). Unlike the FLIPR assay, veratridine and ouabain were both required to facilitate P-CTX-mediated cell death. Cell death was not significant when either veratridine or ouabain were added by themselves (data not shown). These findings are in agreement with related studies [23, 24].

### MTX- and CTX-like Activity in Fractionated *Gambierdiscus* Extracts

**Fig 4:**
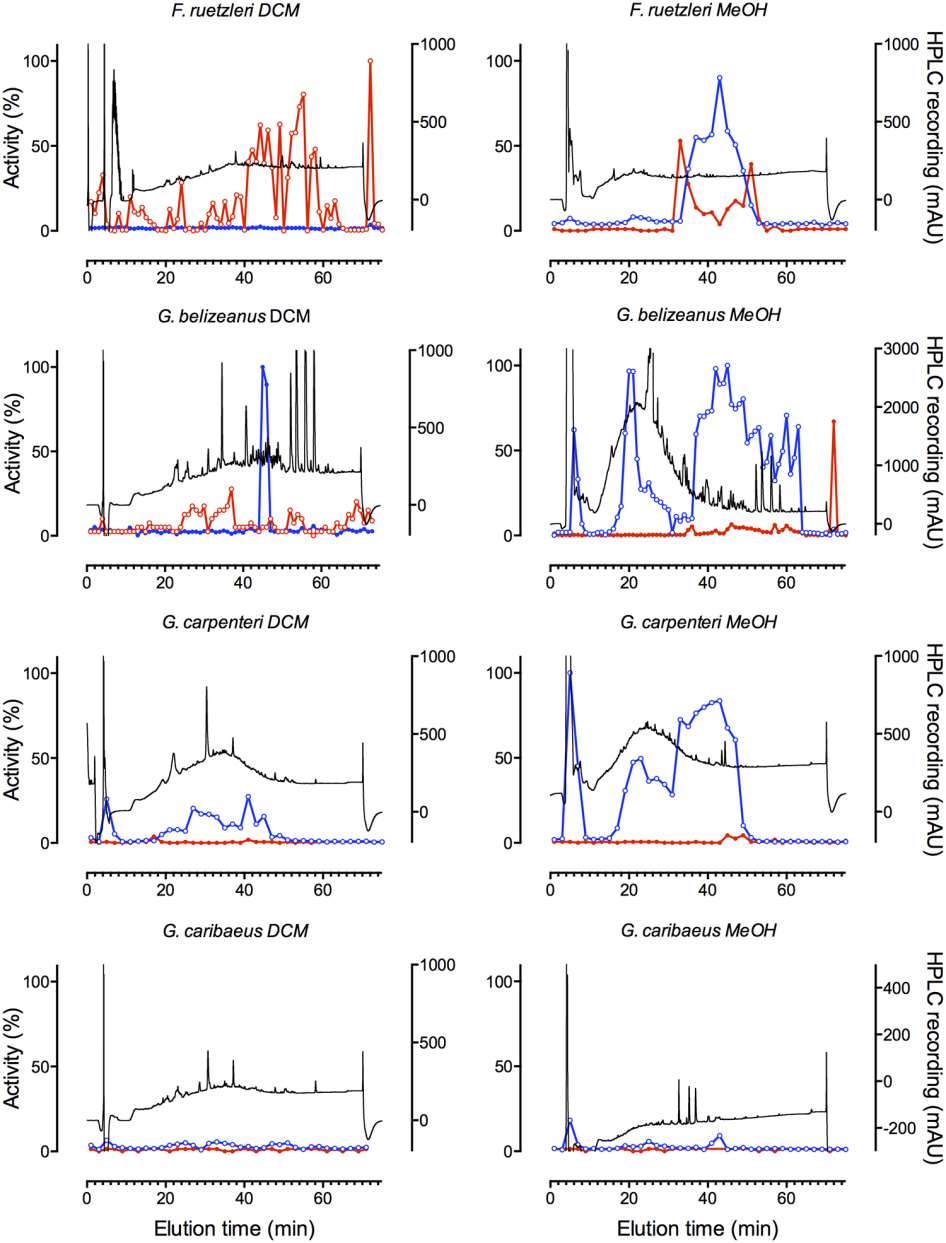
CTX-like and MTX-like activity in HPLC fractionated *Gambierdiscus* and *Fukuyoa* extracts. Dichloromethane (DCM) and methanol (MeOH) extracts of Caribbean *F*. *ruetzleri* (Belize, Gam1), *G*. *belizeanus* (St. Barthelemy Island, CCMP 399), *G*. *carpenteri* (Belize, GT4) and *G*. *caribaeus* (Belize, Gam 19) isolates fractionated by HPLC with UV detection (black trace). Fractions were tested for activity using the FLIPR^TETRA^ assay. CTX-like (red trace) and MTX-like (blue trace) activity was expressed as percent activity (%) relative to the most active MTX and CTX-containing fraction tested in this study.

**Fig 5:**
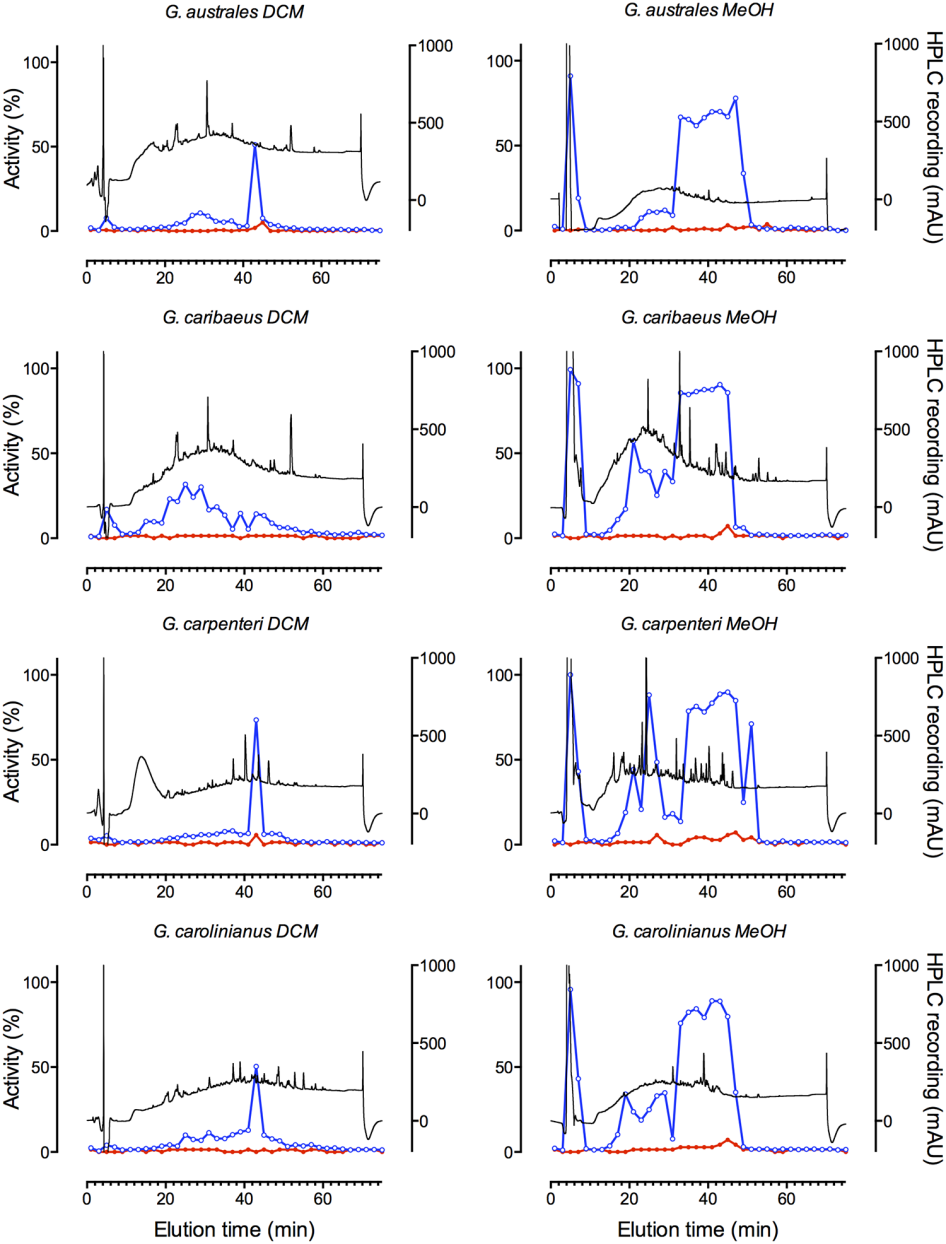
CTX-like and MTX-like activity in HPLC fractionated *Gambierdiscus* extracts of Pacific isolates. Dichloromethane (DCM) and methanol (MeOH) extracts of *G*. *australes* (W B Gam 3), Hawaii *G*. *caribaeus* (Pat HI Jar 2 Gam 2), *G*. *carpenteri* (Pat HI Jar 7 Gam 11), and *G*. *carolinianus* (Pat HI Jar 3 Gam) were fractionated by HPLC with MS UV detection (black trace). Fractions were tested for activity using the FLIPR^TETRA^ assay. CTX-like (red trace) and MTX-like (blue trace) activity was expressed as percent activity (%) relative to the most active MTX and CTX-containing fraction tested in this study.

**Fig 6:**
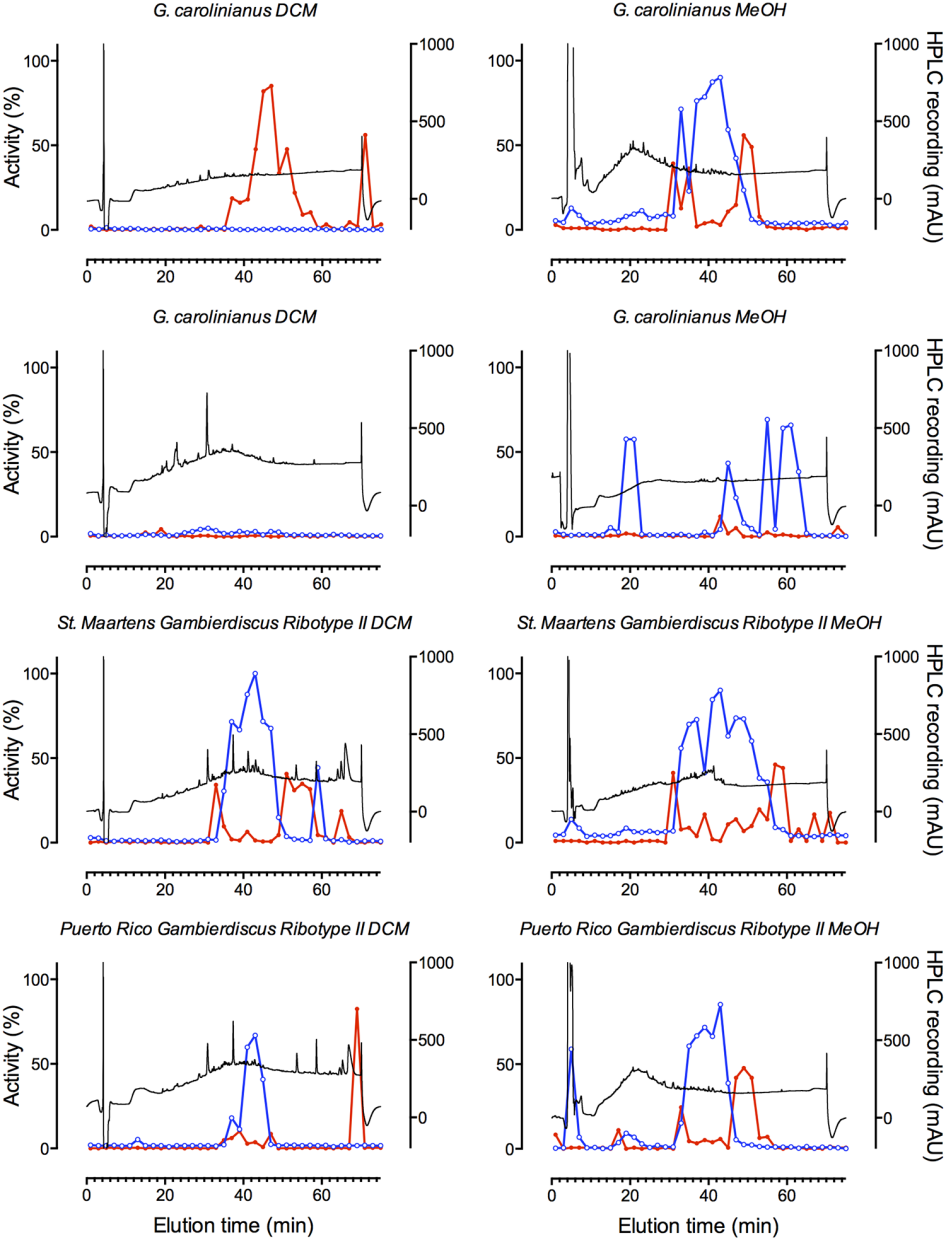
CTX-like and MTX-like activity in HPLC fractionated *Gambierdiscus* extracts from Caribbean/Atlantic isolates. Dichloromethane (DCM) and methanol (MeOH) extracts of Belize *G*. *carolinianus* (Dive 1 Gam 1), North Carolina *G*. *carolinianus* (Kenny 6), St Maartens *Gambierdiscus ribotype II* (St. Maartens Gam 6) and Puerto Rico *Gambierdiscus ribotype II* (Mixed PR Gam 3) were fractionated by HPLC with MS detection (black trace). Fractions were tested for activity using the FLIPR^TETRA^ assay. CTX-like (red trace) and MTX-like (blue trace) activity (%) is given relative to the most active MTX and CTX-containing fraction tested in this study.

*Gambierdiscus* spp. collected from Caribbean and Pacific locations (Table 2) were extracted and the dichloromethane and methanol soluble fractions were separated by HPLC. Using the SH-SY5Y cell assay, MTX-like and CTX-like responses could be clearly identified in multiple fractions (Figs 4–6). Wells receiving MTX-containing fractions exhibited enhanced fluorescence indicative of increased Ca^2+^_i_, which was sustained for the 300 s recording period. As expected, the MTX activity was enriched in the methanol soluble extract, while CTX was enriched in the dichloromethane soluble fraction, consistent with MTX likely being sulfated compared to the uncharged and more hydrophobic CTX. Strong MTX-like activity was detected in all isolates, except a Caribbean isolate of *G*. *caribaeus*, which appeared to produce only trace amounts of MTX (Table 2; Figs 4–6). Of the *Gambierdiscus* spp. tested, only two Caribbean isolates produced prominent levels of CTX-like activity (Fig 4, Table 2). Interestingly, only one of two *G*. *carolinianus* isolates produced CTX, though both produced MTX, while the *F*. *ruetzleri* isolate produced CTX and MTX. In addition, both isolates of *Gambierdiscus* ribotype 2 produced MTX and low levels of CTX-like activity.

Depending on the isolate analyzed, between 1 and 4 separate zones of MTX-like activity were observed in the methanol extracts (Figs 3–5; Table 2). All isolates except one of the *G*. *carolinianus* isolates had MTX-like activity that eluted just after the void volume at ~6 min. For many of the isolates this was one of the dominant toxic fractions. The MTX activity eluting at ~21 min was similarly widely distributed among isolates, but relative to the other active fractions typically showed lower activity. A third and typically dominant toxic zone eluted as a broad peak from 36–46 min and may contain multiple MTXs (Table 2). Interestingly, both Pacific and Caribbean isolates showed activity in each of these zones, suggesting they contain the same or related MTXs. In contrast, a fourth zone of activity eluting at ~80 min was observed in two Caribbean isolates but not the Pacific isolates (Figs 4–6).

Most Caribbean isolates and all Pacific isolates that were tested produced little or no measurable CTX-like activity in the DCM fraction (Figs 4–6; Table 2). Prominent CTX-like activity was detected in *F*. *ruetzleri* eluting at ~45, 55 and 72 min and in *G*. *carolinianus* eluting at ~45 and 72 min. Late eluting activity (~70 min) was also observed in the DCM fractions from *Gambierdiscus* ribotype 2 from Puerto Rico, while *Gambierdiscus* ribotype 2 from St. Maartens had activity eluting at ~55 min. Additional weak activity eluting at the boundary to MTX activity was also observed for Caribbean isolates, activity which may relate to interference associated with specific MTXs. Such interference may explain the appearance of CTX-like activity in some methanol fractions from Caribbean isolates.

For some extracts, MTX-like activity was observed to partition into the DCM fraction and CTX into the methanol fraction (Figs 4–6). Previous work on methonolic extracts in *Gambierdiscus* species using as series of calcium channel pathway inhibitors and ion channel blockers showed that all the observed hemolytic activity was consistent with induction of Ca^2+^ influx by MTX or MTX-like compounds (calcium channel activator toxins) [21]. Based on these data, it is likely that that the fluorescence increased in the SH-SY5Y cells loaded with fluorescent Ca_2+_ dye is the result of Ca^2+^ influx triggered by MTX or MTX-like compounds. Nonetheless, the level of MTX carryover remains to be quantified in future studies. The CTX carry over into the methanolic MTX fraction did not affect the estimate of MTX activity as CTX was incapable of producing a fluorescence change in the absence of veratridine. This carry over was only visible in methanolic cell fractions containing CTX-like compounds, and little or no MTX-like compounds, as a potentiation of veratridine-induced responses (see Fig 6, *G*. *caribaeus* Methanol fraction panel). When MTX-like compounds carried over into some of the DCM fractions, they triggered an immediate increase in fluorescence as soon as the sample was added, thus potentially obscuring the CTX-mediated potentiation of veratridine responses. The result is a potential underestimate of the number of CTX fractions present in extracts where MTX-like activity is present. Subsequent experiments have shown that this carry over can be minimized by increasing the extraction volumes 10-fold (unpublished results).

In many cases, broad peaks (consecutive fractions showing high % activity) in the MeOH and DCM fractions were represented by narrower more well-defined peaks in the corresponding DCM and methanol carryover fractions. These data are consistent with the original broad peak of activity containing more than one structurally related congener.

## Discussion

The SH-SY5Y calcium assay optimised in this study worked well for characterizing CTX-like and MTX-like activity in fractionated extracts from *Gambierdiscus* and *Fukuyoa* cells prepared using a modified ciguatoxin rapid extraction method (CREM) [25]. This approach allowed rapid screening of *Gambierdiscus* samples for MTX-like and CTX-like activity. Using this approach, the number of toxic zones suggested at least one to four MTX were present in the methanol fractions while none to three CTX were detected in the methanol fractions (Table 1). As previously observed, all of the isolates produced at least trace amount of MTX, indicating these compounds likely play an important cellular function [21]. To date, three maitotoxins 1–3 (MTX-1, MTX-2, MTX3) have been identified [26, 27]. MTX-1 and MTX-3 are disulphated polyethers whereas MTX-2 is monosulphated. The degree of sulphation appears to affect toxicity, with the more sulphated congeners typically eluting earlier on reversed-phase HPLC and being more potent [26, 28, 29]. Based on mouse lethality, previously only one MTX has been found to dominate [3]. In contrast, the data from this study indicate that each *Gambierdiscus* isolate produced multiple MTX congeners, as has been observed for ciguatoxins [3, 30]. Differences in *in vitro* and *in vivo* potency may account for the detection of additional MTX congeners using the cell-based assay compared to mouse bioassay. Given that the number of extracted cells was similar (except for *G*. *belizeanus*), and all cells were harvested in log phase growth, the observed variation in the level of activity across equivalent fractions from different isolates indicates high interclonal variation in MTX production. This observation is consistent with the study of Holland et al. [21], which used a red blood cell hemolytic assay to estimate the relative toxicity of 56 *Gambierdiscus* and *Fukuyoa* isolates representing six species. Further work is needed to determine if the levels and types of MTX produced by different isolates is fixed or shifts with nutrient, temperature, salinity and/or growth stages. Interestingly, our data suggest that isolates of the same species from the Pacific are capable of producing similar suites of MTX, albeit with at times remarkable differences in their levels.

Differences in the potential MTX congeners being produced by isolates belonging to genetically different clades within the genus *Gambierdiscus*, or between *Fukuyoa* vs. *Gambierdiscus* isolates, was also examined [6,11]. No consistent clade- or genus-specific fractions were observed. This indicates that the variation in the toxic fractions being produced are isolate-specific rather than species-specific (Table 2). Similarly, no coherent pattern in the toxic MTX fractions produced by Pacific versus Caribbean/Atlantic region isolates was found, except for the late eluting MTX-like activity. Specifically, the two *G*. *carpenteri* isolates from different basins produced nearly identical toxic fractions, whereas the profiles for two *G*. *caribaeus* and two *G*. *carolinianus* isolates varied.

The profiles of the fractionated DCM extracts indicated that 4 of 7 Caribbean isolates and none of the Pacific isolates screened produced significant levels of CTX-like activity. In those isolates producing measurable CTX, the number of potential congeners ranged from 1 to 3, with several showing low levels of additional CTX-like activity. *Fukuyoa ruetzleri* had the greatest number of fractions with CTX-like activity, which mostly overlapped CTX-containing fractions found in *Gambierdiscus* isolates. Given the MTX congeners also overlapped, it appears that species from both genera produce a similar suite of bioactive polyethers and thus both genera both could potentially contribute to ciguatera risk. The multiple CTX congeners found in Caribbean species is reminiscent of the multiple ciguatoxin congeners produced by Pacific *G*. *polynesiensis* clones [30]. The separate activities at ~ 45 and 55 min in *F*. *ruetzleri*, and potentially in *G*. *carolinianus*, may relate to the HPLC-separable C-CTX epimers identified previously in ciguateric Caribbean fish [31, 32]. Interestingly, there was no correlation between the number of CTX and MTX congeners produced by isolates from the same species. For example the Caribbean *G*. *carpenter*i isolate produced at least 3 distinct CTX congeners and no MTX. These data indicate that CTX and MTX are regulated differently and that MTX may play a more central role in *Gambierdiscus* metabolism than CTX [21].

In conclusion, we have streamlined the extraction of polyether toxins from *Gambierdiscus* and related species and developed a new cell-based fluorescence assay that can rapidly identify CTX- and MTX-like activity. Maitotoxin production was observed in 11 of 12 isolates analysed (with trace amounts in the remaining isolate), with 6 of 12 producing at least two forms of maitotoxin. In contrast, only 2 Caribbean isolates produced detectable levels of ciguatoxin-like activity despite a detection limit of >30 pM. Our approach examined clonal dinoflagellate cultures but has potential to be used to assess the ciguatoxin potential of wild collected samples of *Gambierdiscus* and to replace the *in vivo* mouse bioassay for ciguatoxins in fish flesh. The ability to rapidly identify ciguatera-related polyether toxins has implications for the evaluation of ciguatera risk associated with *Gambierdiscus* and related species.

## Abbreviations

CTX: Ciguatoxin
MTX: maitotoxin
LC: liquid chromatography
MS: mass spectrometry
CREM: ciguatoxin rapid extraction method
FLIPR: fluorescent imaging plate reader
